# Short- and Mid-Term Outcomes in Patients Deemed Inoperable Undergoing Transapical and Transfemoral TAVR with an STS-PROM below Four Percent

**DOI:** 10.3390/jcm10132993

**Published:** 2021-07-05

**Authors:** Verena Veulemans, Katharina Hellhammer, Armin Borhan Azad, Shouheng Goh, Christian Drake, Oliver Maier, Kerstin Piayda, Amin Polzin, Arash Mehdiani, Christian Jung, Ralf Westenfeld, Malte Kelm, Artur Lichtenberg, Tobias Zeus

**Affiliations:** 1 Division of Cardiology, Pulmonology and Vascular Medicine, Medical Faculty, Heinrich Heine University, Moorenstr. 5, 40225 Düsseldorf, Germany; Armin.BorhanAzad@med.uni-duesseldorf.de (A.B.A.); Shouheng.Goh@med.uni-duesseldorf.de (S.G.); chdra100@hhu.de (C.D.); Oliver.Maier@med.uni-duesseldorf.de (O.M.); Kerstin.Piayda@med.uni-duesseldorf.de (K.P.); amin.polzin@med.uni-duesseldorf.de (A.P.); christian.jung@med.uni-duesseldorf.de (C.J.); ralf.westenfeld@med.uni-duesseldorf.de (R.W.); malte.kelm@med.uni-duesseldorf.de (M.K.); zeus@med.uni-duesseldorf.de (T.Z.); 2 Cardiology and Vascular Medicine, Contilia Herz-und Gefäßzentrum, 45138 Essen, Germany; k.hellhammer@contilia.de; 3 Division of Cardiovascular Surgery, Medical Faculty, Heinrich Heine University, Moorenstr. 5, 40225 Düsseldorf, Germany; arash.mehdiani@med.uni-duesseldorf.de (A.M.); artur.lichtenberg@med.uni-duesseldorf.de (A.L.); 4 Cardiovascular Research Institute Düsseldorf (CARID), Moorenstr. 5, 40225 Düsseldorf, Germany

**Keywords:** TAVR, TAVI, transfemoral, transapical, outcome

## Abstract

Transapical (TA) TAVR is known to be associated with increased mortality and vascular complications compared with transfemoral (TF) TAVR in high-risk and inoperable patients. However, safe alternative access methods remain crucial. We aimed to (1) evaluate the 30-day and 1-year outcomes comparing TA and TF TAVR in patients with an STS-PROM of <4% deemed inoperable and (2) determine dependent and independent predictors for all-cause one-year mortality. Data were collected from a single-center registry consisting of 340 eligible patients. One-to-one propensity score matching was performed (*n* = 50 TA, *n* = 50 TF). Primary endpoints were all-cause mortality, stroke, and major bleeding. Predictors for all-cause one-year mortality were evaluated. Thirty-day mortality (TF vs. TA: 0.0% vs. 4.0%; *p* = 0.153) was comparable in both cohorts. One-year all-cause mortality was twice as high in TA patients (TF vs. TA: 10.0% vs. 20.0%, p logrank = 0.165, HR 2.10). Cerebrovascular events and major bleeding during one-year follow-up were similar. The multivariate analysis identified hemoglobin <12 g/dL at admission and dual antiplatelet therapy as strong predictors for one-year mortality. Although femoral access is the primary access with favorable 30-day and 1-year results, transapical access was successful for patients unsuitable for TF TAVR, showing acceptable short- and mid-term results in inoperable patients with low-risk profiles.

## 1. Introduction

In the past, transapical (TA) transcatheter aortic valve replacement (TAVR) was often perceived to be associated with increased mortality and enhanced vascular complications, according to the underlying high-risk profile, compared with transfemoral (TF) TAVR [1,2]. Although most patients can be treated with TF TAVR today using smaller delivery sheaths and newer-generation valves, up to one-third of eligible patients may not be suitable for this approach [3]. With the expansion of TAVR in low-risk patients [4], safe alternative access methods remain crucial for patients without adequate transfemoral access. Currently, there are no research data regarding the outcomes of formally low-risk patients undergoing transapical compared with transfemoral TAVR who were deemed inoperable due to specific individual reasons. Therefore, we aimed to (1) evaluate the 30-day and 1-year outcomes comparing TA and TF TAVR in inoperable patients with an STS-PROM of <4% using newer-generation prostheses and (2) determine dependent and independent predictors for all-cause one-year mortality.

## 2. Materials and Methods

Study population: Among 1711 consecutive patients with symptomatic severe aortic stenosis (AS) who underwent either transfemoral (TF) or transapical (TA) TAVR with newer-generation self-expandable devices (SAPIEN 3, Edwards Lifesciences; Evolut R/Pro platform, Medtronic) from 2014 to December 2019 at the Heart Centre Düsseldorf, we included 340 patients with an STS-PROM of <4% and complete datasets in this retrospective analysis. All patients were deemed (relatively or absolute) inoperable by the heart team due to previous surgery, frailty, advanced aortic calcifications, pulmonary disease, high bleeding risk, or cancer. The main reasons to conduct the transapical access were advanced peripheral artery disease, a critical aortic arch morphology, and allocation to TA based on the patient’s request. Frailty was defined as a reduced stage of health, including falls, incident disability or immobilization, slowed walking speed, low physical activity, and or unintentional weight loss. All procedures were performed according to the current guideline recommendations and under local (TF) or general (TA) anesthesia. The initial study cohort was further separated into patients undergoing TF (*n* = 290; 85.3%) or TA TAVR (*n* = 50; 14.7%). Due to the heterogeneity of the two populations, propensity score matching was employed to match TF and TA patients for age, gender, previous CABG, pulmonary hypertension, peripheral artery disease, and porcelain aorta. One-to-one propensity score matching created 50 patients in each cohort for a total of 100 patients of the final study cohort. Please see the overview of this study in Supplementary Figure S1. All patients provided written informed consent for TAVR and the use of their clinical, procedural, and follow-up data in research. The study procedures were conducted in accordance with the Declaration of Helsinki, and the institutional Ethics Committee of Heinrich-Heine University approved the study protocol (4080). The study was registered (NCT01805739).

Study endpoints: Primary endpoints of this study were all-cause mortality, stroke, and major bleeding after 30 days and 1 year. Secondary endpoints were defined according to the VARC-2 definitions [5].

Statistical analysis: The collected data included patient characteristics, imaging findings, periprocedural in-hospital data, laboratory results, and follow-up data. Continuous data are described by the mean and standard deviation or median and upper and lower 95% confidence interval (interquartile ranges), and categorical variables are described by frequencies and percentages. Continuous variables were compared using a Student’s t-test or the Kolmogorov–Smirnov test, depending on the variable distribution in a heterogeneous sample size. Categorical variables were compared using Fisher’s exact test. One-to-one propensity score matching was realized according to existing statistical guidelines based on the heterogeneity of the overall population of the study. Binomial multivariate regression was performed to assess independent predictors of one-year mortality. Covariates associated with one-year mortality in the univariate analysis (*p* < 0.1) were entered into the multivariate model. Model discrimination accuracy was evaluated using ROC analysis and the C-index (area under the curve). The data analysis was performed using the statistical software SPSS (version 27.0.1, SPSS Inc., Chicago, IL, USA), GraphPad Prism (version 6.0, GraphPad Software, San Diego, CA, USA), and Wizard 2 Statistics & Analysis (Evan Miller). All statistical tests were two-tailed, and a value of *p* < 0.05 was considered statistically significant.

## 3. Results

### 3.1. Baseline Characteristics

Baseline characteristics did differ according to the particular risk profile in the different access routes. Patients undergoing TA TAVR were predominantly male, younger, had more previous CABG, and suffered more peripheral artery disease and porcelain aorta but less pulmonary hypertension. A full overview of the baseline clinical and functional characteristics is displayed in Supplementary Table S1.

One-to-one propensity score matching created 100 patients (TF = 50 and TA = 50) with an equivalent risk profile to evade the apparent selection bias. The two propensity-matched groups were more balanced according to their baseline characteristics but still differed concerning gender (TF vs. TA: male 54.0% vs. 82.0%; *p* = 0.003 *) and frailty condition (TF vs. TA: 50.0% vs. 26.0%; *p* = 0.013 *). A full overview of the baseline clinical and functional characteristics is displayed in Table 1. All further analyses were established in the propensity-matched cohorts.

### 3.2. General Procedural Characteristics

Procedural details and clinical outcomes are displayed in Table 2. Contrast use (TF vs. TA: 143.0 ± 62.5 mL vs. 93.0 ± 28.9 mL; *p* < 0.001 *), fluoroscopy time (TF vs. TA: 20.3 ± 5.5 min vs. 8.1 ± 5.0 min; *p* < 0.001 *), and dose area product (TF vs. TA: 7.429 Gyx ± 4.211 cm^2^ vs. 3.012 Gyx ± 2.491 cm^2^; *p* < 0.001 *) were lower in the TA cohort. Predilatation was less frequently observed in TF patients (TF vs. TA: 82.0% vs. 98.0%; *p* = 0.008 *) whereas postdilatation was only necessary in the TF cohort (TF vs. TA: 12.0% vs. 0.0%; *p* = 0.012 *). All intraprocedural complications were comparable between both cohorts.

### 3.3. Thirty-Day Outcome and Functional Status

Thirty-day mortality was very low in TF and mildly enhanced in TA patients (TF vs. TA: 0.0% vs. 4.0%; *p* = 0.153). Cerebrovascular events were similar in both cohorts (4.0%), whereas major bleeding was twice as high in the TA cohort (8.0%). TA patients showed a prolonged in-hospital stay (TF vs. TA: 13.3 ± 7.0 days vs. 16.3 ± 7.5 days; *p* = 0.042 *), mostly driven by a prolonged ICU stay (TF vs. TA: 3.9 ± 4.1 days vs. 6.5 ± 3.6 days; *p* = 0.001 *). For further information, please see Table 3. Functional improvement was observed in both groups without differences concerning prosthesis function and paravalvular regurgitation as evaluated by the pre-discharge echocardiography.

### 3.4. One-Year Clinical Outcome

**Figure 1:**
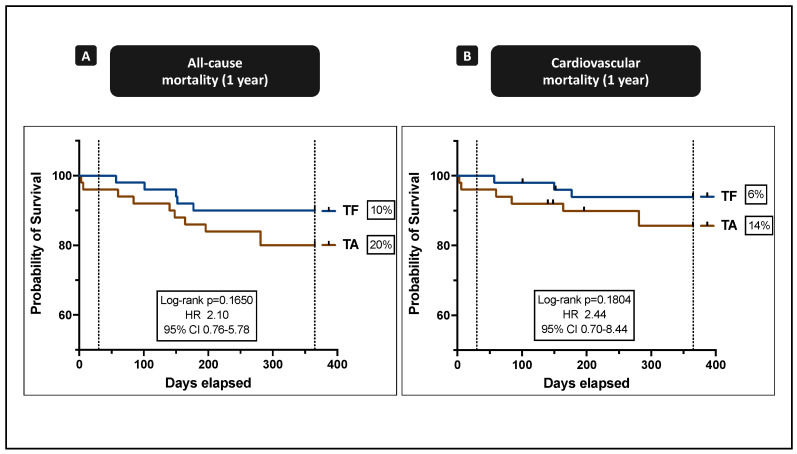
All-cause and cardiovascular mortality at one year. The percentage value on the right axis corresponds to the mortality rate. (**A**) All-cause mortality comparing TF and TA TAVR. (**B**) Cardiovascular mortality comparing TF and TA TAVR.

One-year all-cause mortality was high in both cohorts and twice as high in TA patients (Figure 1A; TF vs. TA: 10.0% vs. 20.0%; p_logrank = 0.165; HR 2.10; 95%-CI = 0.76–5.78). One-year cardiovascular mortality was mediocre in both cohorts but was also twice as high in TA patients (Figure 1B; TF vs. TA: 6.0% vs. 14.0%; p_logrank = 0.180; HR 2.44; 95%-CI = 0.70–8.44). One death due to chronic myelogenous leukemia was documented in a TA patient in whom the TAVR was combined with MIDCAB and PCI. In detail, causes of overall mortality after 1 year were cancer (*n* = 3; 20.0%) and comorbidities leading to all-cause death (*n* = 1; 6.7%). Overall cardio- and cerebrovascular death was documented in 11 cases (73.3%).

**Figure 2:**
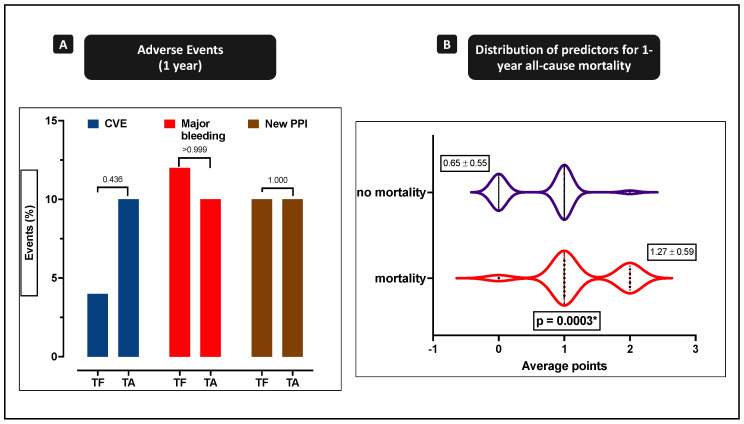
One-year outcome and independent predictors for one-year mortality. (**A**) Cerebrovascular events (CVE), major bleeding, and need for permanent pacemaker implantation (PPI) during one-year follow-up were comparable in both cohorts. (**B**) Distribution of independent predictors for one-year mortality: patients that died from any cause showed a higher summation of the predictors (1.3 ± 0.6 vs. 0.7 ± 0.6; *p* < 0.001 *). * *p* < 0.05.

After one year, cerebrovascular events (TF vs. TA: 4.0% vs. 10.0%; *p* = 0.436) were also numerically enhanced in the TA cohort. Major bleeding during one-year follow-up (TF vs. TA: 12.0% vs. 10.0%; *p* > 0.999) was comparable in both cohorts. Interestingly, the need for permanent pacemaker implantation (PPI; TF vs. TA: 10.0% vs. 10.0%; *p* = 1.000) was the same in both cohorts (Figure 2A). The functional improvement as assessed by NYHA stage during one-year follow-up was similar in TF and TA patients (Supplementary Figure S2).

Dependent predictors of one-year mortality were determined to be disabling bleeding (OR 9.94 (1.96–50.42), *p* = 0.006 *), moderate-to-severe tricuspid regurgitation (OR 5.06 (1.01–25.46), *p* = 0.049 *), moderate-to-severe multivalvular disease (OR 2.88 (0.90–9.24), *p* = 0.076), urgent TAVR (OR 2.59 (0.85–7.90), *p* = 0.094), low red blood cell count (OR 3.97 (1.28–12.36), *p* = 0.017 *) at admission, severe acute kidney injury (OR 10.38 (1.57–68.60)), *p* = 0.015 *), and dual antiplatelet therapy (OR 0.27 (0.08–0.91), *p* = 0.034 *) at discharge. Apart from other relevant baseline parameters, the TA approach was not confirmed as a dependent risk factor by the univariate analysis (OR 2.25 (0.71–7.14), *p* = 0.169). In the multivariate analysis, only hemoglobin <12 g/dL at admission and the use of dual antiplatelet therapy were identified as independent predictors for one-year mortality. The C-statistic revealed a mediocre association of the covariates mentioned above with mortality (Table 4: AUC = 0.75; 95% CI = 0.61–0.88; *p* = 0.0024 *). Patients that died from any cause showed a higher summation of the predictors (Figure 2B; 1.3 ± 0.6 vs. 0.7 ± 0.6; *p* < 0.001 *).

## 4. Discussion

In the past, studies have frequently reported worse outcomes comparing transapical to transfemoral TAVR in patients at high surgical risk. However, safe alternative access techniques remain crucial for patients without adequate transfemoral access. To our knowledge, this is the first real-world study comparing short- and mid-term outcomes in transapical and transfemoral TAVR concerning patients that were deemed inoperable by the heart team but had an STS-PROM below four percent. The main readouts of our retrospective study revealed that:

1. Mortality after 30 days was only mildly enhanced in TA patients and twice as high after one year.
2. Cerebrovascular events, major bleeding, and even pacemaker need were nearly similar during a one-year follow-up in TF and TA patients.
3. Other factors besides transapical access were identified as independent predictors for one-year mortality in low-risk patients (hemoglobin < 12 g/dL at admission and use of dual antiplatelet therapy).

### 4.1. Alternative Access Sites

In many centers, the transapical approach is still considered the second choice if transfemoral access is not suitable, but other access sites are expanding in number and expertise [6]. Alternative access sites include the transaortic, axillary/subclavian, brachiocephalic, transcarotid, and transcaval approaches, which have different advantages, disadvantages, and outcomes. In this study, the main reasons to tailor patients to TA TAVR were significant peripheral artery disease at the level of the common femoral arteries and porcelain aorta.

### 4.2. Procedural Characteristics

We observed several intraprocedural differences due to the nature of the access site. Contrast medium use, fluoroscopic time, and dose area product were significantly reduced in the TA cohort. This is in line with the transapical approach’s well-known advantages, including less contrast and less fluoroscopy time due to the short distance from the sheath to the annulus [3,7]. Postdilatation was only necessary for TF patients, taking the use of self-expandable devices into account. All intraprocedural complications were comparable and generally low. Device success was 100% in patients undergoing TF TAVR and 99% in TA patients.

### 4.3. Thirty-Day Outcome

Regarding the primary endpoint, 30-day mortality was very low in TF (0.0%) and mildly enhanced in TA patients at 4% (*p* = 0.153). In a comprehensive comparison of multicenter registries and randomized control trials for TAVR [8], pooled 30-day mortality rates were 6.8% in the TF group compared with 3.9% in the PARTNER-TF cohort. In the TA groups, registry cohorts showed 30-day mortality rates of 12.2% compared with 3.8% in the PARTNER TA group. Thus, the pooled 30-day mortality from the registries was significantly higher than in the PARTNER trial. This phenomenon may have been driven by rigorous patient screening and high-volume load/expertise within the trial sites, dismissing real-world treatment and outcomes. However, real-world data also could have been improved by centers performing high-volumes of TA TAVR [9], resulting in 30-day mortality decreasing to 4.2% in later years [10], suggesting that practice experience contributes to favorable outcomes.

Cerebrovascular events were similar in both cohorts at 4.0%, whereas major bleeding was twice as high in the TA cohort (8.0%) but without statistical significance. This is in line with the European SOURCE registry data that reported a greater incidence of major bleeding among patients undergoing TA TAVR [11]. All other VARC-2-related adverse events were also statistically comparable. However, TA patients showed a prolonged in-hospital stay, probably mainly driven by a prolonged ICU stay due to the procedure’s invasiveness and the need for temporary ventilation. The invasiveness is the most discussed disadvantage of this access site, potentially leading to enhanced morbidity and mortality in a particular group of patients. In this context, it must be mentioned that the antithrombotic regime may have an impact on bleeding, morbidity, and mortality. In this study, most of the TF patients were discharged with dual antiplatelet therapy (62%), whereas many TA patients had or developed an indication for oral anticoagulation and were discharged with single or combined N/OAC therapy (52%). The optimal antithrombotic regimen following TAVI has yet to be determined and is currently the focus of several randomized trials, which aim to balance bleeding and thrombotic risk in the near future [12].

### 4.4. One-Year Clinical Outcome and Independent Predictors for All-Cause One-Year Mortality

One-year all-cause mortality was high in both cohorts and twice as high in TA patients (10.0% vs. 20.0%), but statistically comparable, probably due to the limited case number. One-year cardiovascular mortality was mediocre in both cohorts, but also twice as high in TA patients (6.0% vs. 14.0%). Ancient pooled one-year mortality was 20.8% in the TF group compared with 26.2% in the PARTNER-TF cohort [8]. In the TA groups, registry cohorts showed one-year mortality rates of 32.2% compared with 29.0% in the PARTNER TA group. As a result of heterogenous risk-profiles of the cohorts, outcomes between TF and TA TAVR were reported to range from similar to divergent in other registries [13,14,15]. However, our results show that one-year mortality was similarly improved in patients with an STS-PROM below four percent.

Cerebrovascular events after one year were also numerically enhanced in the TA cohort without statistical difference. Consistent with other studies, we found no difference between TF and TA TAVR concerning stroke. Thus, the theoretical benefit of TA TAVR in avoiding manipulation of the aorta through direct access did not translate into a reduction in cerebrovascular events [16,17,18]. Major bleeding and the need for a permanent pacemaker need during one-year follow-up were also identical and mediocre in both cohorts.

Dependent predictors of one-year all-cause mortality were determined to be hemoglobin <12 g/dL at admission, as identified by Youden’s Index/ROC analysis, and the use of dual antiplatelet therapy. As dual antiplatelet therapy was a protective factor in the prediction of one-year mortality, an inverse calculation as a risk profile was established in which patients that died from any cause showed a higher summation of the (inverse) predictors (1.3 ± 0.6 vs. 0.7 ± 0.6; *p* < 0.001 *), supposing that other risk factors contributed to the higher mortality of TA patients, probably driven by enhanced bleeding risk. Neither the access site nor other clinical or procedural characteristics had an impact on the outcomes in this study.

### 4.5. Current Knowledge on One-Year Mortality in Low-Risk Patients Using Alternative Access Routes

Comparing our real-world results with the current low-risk data on TF TAVR, one-year mortality was higher (10%) than that in the randomized trials, including the PARTNER III (1.0%) [19], Evolut Low-risk (2.4%) [20], and NOTION (4.9%) trials [21]. However, as our patients were deemed relatively or absolute inoperable due to previous surgery, frailty, advanced aortic calcifications, pulmonary disease, high bleeding risk, or cancer, the results cannot be compared to classical low-risk cohorts. As mentioned before, the type of patient screening within randomized trials may also lead to a difference in real-world outcomes. Furthermore, the presence of multivalvular disease was high, approximately 20%, which might have led to advanced secondary right heart failure. Although there are no current data on outcomes in real low-risk patients using different access routes, some studies showed favorable and comparable results between TF and non-TF access sites in all-comer patients [22], strengthening the argument that different approaches can be tailored to a patient-specific risk profile. However, to our knowledge, this is the first study that compared short- and mid-term outcomes in patients with an STS-PROM below four percent using the two leading access routes.

### 4.6. Limitations

This study is a single-center, retrospective analysis with associated unavoidable limitations due to its design. Obviously, the power of this study is limited because of the case number, but it is empowered by propensity-matched score analysis. Thus, the results of this study concerning 30-day and one-year outcomes can only be considered as trends.

## 5. Conclusions

Although femoral access is the primary access and has favorable 30-days and 1-year results, transapical access was proven to be safe and successful for patients unsuitable for TF TAVR, showing acceptable short- and mid-term results in inoperable patients with an STS-PROM of <4%. National registry and multicenter data are needed to validate these observed trends.

## Figures and Tables

**Table 1:** Patient clinical and functional characteristics (propensity score-matched cohort).

Clinical Data	Overall (*n* = 100)	TF (*n* = 50)	TA (*n* = 50)	*p*-Value
Age, years	74.5 ± 8.3	75.7 ± 8.3	73.4 ± 8.3	0.180
Gender, male	68 (68.0)	27 (54.0)	41 (82.0)	0.003 *
BMI	28.0 ± 4.6	28.3 ± 4.6	27.7 ± 4.6	0.556
CAD	72 (72.0)	35 (70)	37 (74.0)	0.656
Previous PCI	31 (31.0)	16 (32.0)	15 (30.0)	0.829
Previous CABG	23 (23.0)	10 (20.0)	13 (26.0)	0.476
Previous valve	4 (4.0)	2 (4.0)	2 (4.0)	1.000
Previous PPI	12 (12.0)	6 (12.0)	6 (12.0)	1.000
Preexisting LBBB/RBBB	5 (5.0)	3 (6.0)	2 (4.0)	0.646
Preexisting AVB	2 (2.0)	1 (2.0)	1 (2.0)	1.000
Arterial hypertension	94 (94.0)	46 (92.0)	48 (96.0)	0.400
PHT	54 (54.0)	29 (58.0)	25 (50.0)	0.422
Diabetes mellitus	29 (29.0)	14 (28.0)	15 (30.0)	0.826
PAD	49 (49.0)	20 (40.0)	29 (58.0)	0.072
CVD	24 (24.0)	15 (30.0)	9 (18.0)	0.160
Porcelain aorta	23 (23.0)	11 (22.0)	12 (24.0)	0.812
Hostile Aorta	14 (14.0)	6 (12.0)	8 (16.0)	0.564
Previous RRT	0 (0.0)	0 (0.0)	0 (0.0)	1.000
CKD	36 (36.0)	21 (42.0)	15 (30.0)	0.211
COPD	23 (23.0)	10 (20.0)	13 (26.0)	0.476
Frailty	38 (38.0)	25 (50.0)	13 (26.0)	0.013 *
**Functional Data**				
STS score, %	2.7 ± 0.8	2.8 ± 0.7	2.5 ± 0.9	0.075
HAS-BLED score	2.9 ± 1.0	2.9 ± 0.9	2.8 ± 1.0	0.421
LVEF, %	50.4 ± 11.6	46.3 ± 10.7	52.7 ± 11.7	0.194
AVA, cm^2^	0.8 ± 0.2	0.8 ± 0.2	0.8 ± 0.2	0.318
dPmax, mmHg	63.7 ± 21.1	61.9 ± 18.2	65.7 ± 23.9	0.424
dPmean, mmHg	39.5 ± 15.5	36.2 ± 12.7	43.2 ± 17.7	0.043 *
NYHA III/IV	64 (64.0)	31 (62.0)	33 (66.0)	0.677

* *p* < 0.05; values are means ± SD, medians ± interquartile range, or *n* (%). AVA, aortic valve area; AVB, atrioventricular block; BMI, body mass index; CABG, coronary artery bypass graft; CAD, coronary artery disease; COPD, chronic obstructive pulmonary disease; CVD, cerebrovascular disease; dPmean/max, mean/max. transvalvular gradient; LBBB, left bundle branch block; LVEF, Left ventricular ejection fraction; PCI, percutaneous coronary intervention; PHT, pulmonary hypertension; PAD, peripheral artery disease; PPI, permanent pacemaker implantation; RBBB, right bundle branch block; RRT, renal replacement therapy.

**Table 2:** Procedural characteristics (propensity score-matched cohort).

Procedural Data	Overall (*n* = 100)	TF (*n* = 50)	TA (*n* = 50)	*p*-Value
Sapien 3^TM^	76 (76.0)	26 (52.0)	50 (100.0)	<0.001 *
CoreValve Evolut R/Pro^TM^	24 (24.0)	24 (48.0)	0 (0.0)	<0.001 *
Valve-in-Valve	3 (3.0)	2 (4.0)	1 (2.0)	0.558
Bicuspid Valve	1 (1.0)	1 (2.0)	0 (0.0)	0.315
+MIDCAB	2 (2.0)	0 (0.0)	2 (4.0)	0.153
+PCI	1 (1.0)	1 (2.0)	1 (2.0)	0.315
Contrast, mL	118.0 ± 54.6	143.0 ± 62.5	93.0 ± 28.9	<0.001 *
Fluoroscopy time, min	14.3 ± 8.1	20.3 ± 5.5	8.1 ± 5.0	<0.001 *
Dose Area Product, Gyx, cm^2^	5.289 ± 4.117	7.429 ± 4.211	3.012 ± 2.491	<0.001 *
Predilatation	90 (90.0)	41 (82.0)	49 (98.0)	0.008 *
Postdilatation	6 (6.0)	6 (12.0)	0 (0)	0.012 *
**Intraproced. Complications**				
Immediate stroke	1 (1.0)	1 (2.0)	0 (0)	0.315
Aortic dissection	0 (0)	0 (0)	0 (0)	1.000
Annulus rupture	0 (0)	0 (0)	0 (0)	1.000
Coronary obstruction	0 (0)	0 (0)	0 (0)	1.000
Vascular complications	11 (11.0)	8 (16.0)	3 (6.0)	0.110
Valve dislocation	1 (1.0)	0 (0)	1 (2.0)	0.315
Conversion to surgery	0 (0)	0 (0)	0 (0)	1.000
Need of 2nd valve	0 (0)	0 (0)	0 (0)	1.000
Tamponade	0 (0)	0 (0)	0 (0)	1.000
CPR	0 (0)	0 (0)	0 (0)	1.000
Immediate procedural death	1 (1.0)	0 (0.0)	1 (2.0)	0.315
Heart rhythm disturbances	2 (2.0)	1 (2.0)	1 (2.0)	1.000

* *p* < 0.05; values are means ± SD, medians ± interquartile range, or *n* (%). CPR, cardiopulmonary resuscitation; MIDCAB, minimal invasive direct coronary arterial bypass surgery.

**Table 3:** Postprocedural outcomes.

Postprocedural Outcome	Overall (*n* = 100)	TF (*n* = 50)	TA (*n* = 50)	*p*-Value
30-day mortality	2 (2.0)	0 (0.0)	2 (4.0)	0.153
Disabling bleeding	7 (7.0)	3 (6.0)	4 (8.0)	0.695
Major bleeding	6 (6.0)	2 (4.0)	4 (8.0)	0.400
Major vascular complications	14 (14.0)	6 (12.0)	8 (16.0)	0.564
Stroke/TIA	4 (4.0)	2 (4.0)	2 (4.0)	1.000
AKI I-III	15 (15.0)	7 (14.0)	8 (16.0)	0.736
AKI I	10 (10.0)	4 (8.0)	6 (12.0)	0.505
AKI II	0 (0.0)	0 (0.0)	0 (0.0)	1.000
AKI III	5 (5.0)	3 (6.0)	2 (4.0)	0.646
New RRT	5 (5.0)	3 (6.0)	2 (4.0)	0.646
Sepsis	2 (2.0)	2 (4.0)	0 (0.0)	0.153
Endocarditis	0 (0.0)	0 (0.0)	0 (0.0)	1.000
New LBBB/RBBB	15 (15.0)	10 (20.0)	5 (10.0)	0.262
New AVB	6 (6.0)	3 (6.0)	3 (6.0)	1.000
New PPI	5 (5.0)	2 (4.0)	3 (6.0)	0.646
In-hospital stay	14.8 ± 7.4	13.3 ± 7.0	16.3 ± 7.5	0.042 *
ICU stay	5.2 ± 4.1	3.9 ± 4.1	6.5 ± 3.6	0.001 *
**Functional Data at Discharge**				
Vmax (m/s)	2.2 ± 0.4	2.2 ± 0.4	2.2 ± 0.4	0.931
dPmean (mmHg)	11.1 ± 3.1	10.6 ± 3.8	11.8 ± 3.3	0.184
AR ≥ II° (PVL)	4 (4.0)	2 (4.0)	2 (4.0)	1.000
MR ≥ II°	12 (12.0)	4 (8.0)	8 (16.0)	0.218
TR ≥ II°	7 (7.0)	1 (2.0)	6 (12.0)	0.050
MV disease ≥ II°	22 (22.0)	9 (18.0)	13 (26.0)	0.334
**Medication at Discharge**				
DPT	53 (53.0)	31 (62.0)	22 (44.0)	0.071
N/OAC mono	12 (12.0)	3 (6.0)	9 (18.0)	0.065
N/OAC + SPT	20 (20.0)	10 (20.0)	10 (20.0)	1.000
Triple therapy	9 (9.0)	2 (4.0)	7 (14.0)	0.081

* *p* < 0.05; values are means ± SD, medians ± interquartile range, or n (%). AKI, acute kidney injury; AR, aortic regurgitation; DPT, dual antiplatelet therapy; ICU, intensive care unit; MR, mitral regurgitation; MV, multivalvular disease; N/OAC, (new) oral anticoagulants; PPI, permanent pacemaker therapy; PVL, paravalvular leakage; RRT, renal replacement therapy; SPT, single antiplatelet therapy; TIA, transient ischemic attack; TR. tricuspid regurgitation.

**Table 4:** Univariate and multivariate regression analysis of 1-year mortality.

	Univariate Analysis		Multivariate Analysis		ROC		
(A) Risk Factors	OR (95%-CI)	*p*-Value	OR (95%-CI)	*p*-Value	AUC	95%-CI	*p*-Value
Disabling bleeding	9.94 (1.96–50.42)	0.006 *	-	-	0.75	0.61–0.88	0.0024 *
TR ≥ II	5.06 (1.01–25.46)	0.049 *	-	-			
MV disease ≥ II	2.88 (0.90–9.24)	0.076	-	-			
Urgent TAVR	2.59 (0.85–7.90)	0.094	-	-			
Hemoglobin < 12 g/dL	3.97 (1.28–12.36)	0.017 *	8.14 (1.88–35.20)	0.005 *			
AKI (stage 3)	10.38 (1.57–68.60)	0.015 *					
**(B) Protective Factors**							
DPT	0.27 (0.08–0.91)	0.034 *	0.09 (0.02–0.43)	0.003 *			

* *p* < 0.05; values are means ± SD, medians ± interquartile range, or n (%).

## Data Availability

The data presented in this study are available within the article or supplementary material.

## Supplementary Materials

The following are available online at https://www.mdpi.com/article/10.3390/jcm10132993/s1. Figure S1: Flowchart of the study; Figure S2: Functional improvement during 1-year follow-up (FU). Table S1: Patient clinical and functional characteristics.
