# Supplementary material for: Short- and Mid-Term Outcomes in Patients Deemed Inoperable Undergoing Transapical and Transfemoral TAVR with an STS-PROM below Four Percent
Source: J Clin Med. 2021 Jul 5;10(13):2993. doi: 10.3390/jcm10132993 (PMC8267718; doi:10.3390/jcm10132993)
Supplement: Supplementary file 1 [file jcm-10-02993-s001.zip › jcm-1242371-supplementary.pdf]

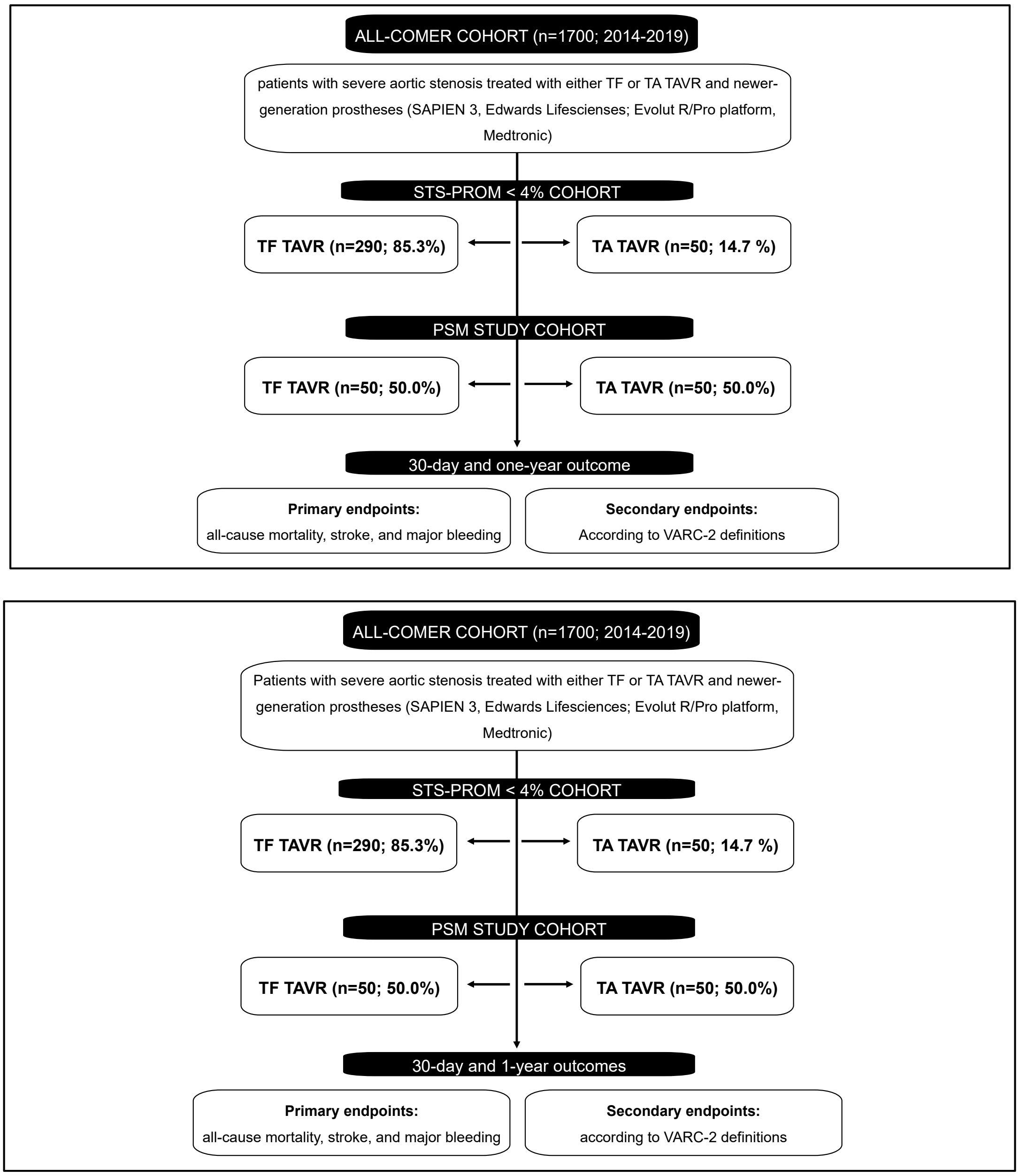

Figure S1. Flowchart of the study; PSM, propensity score matching.

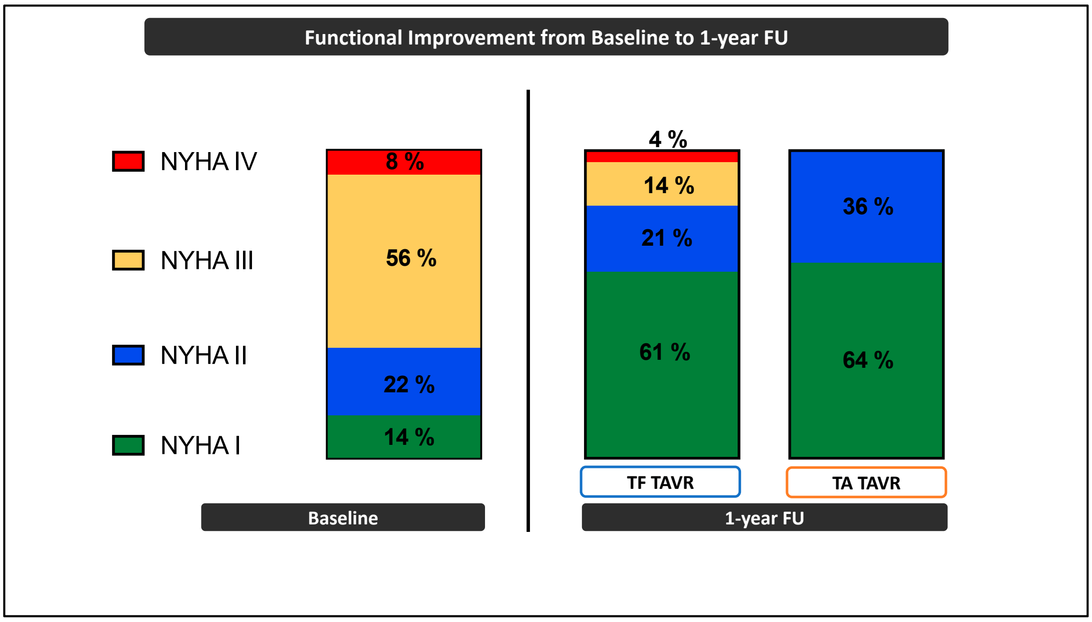

Figure S2. Functional improvement during 1-year follow-up (FU).

Table S1. Patient clinical and functional characteristics (propensity score-matched cohort).

Clinical Data	Overall (n = 340)	TF (n = 290)	TA (n = 50)	p-Value
Age, years	77.4 ± 7.2	78.1 ± 6.8	73.4 ± 8.3	<0.001 *
Gender, male	202 (59.4)	161 (55.5)	41 (82.0)	<0.001 *
BMI	27.7 ± 4.9	27.7 ± 5.0	27.7 ± 4.6	0.986
CAD	230 (67.7)	193 (66.6)	37 (74.0)	0.298
Previous PCI	104 (30.6)	89 (30.7)	15 (30.0)	0.922
Previous CABG	48 (14.1)	35 (12.1)	13 (26.0)	0.009 *
Previous valve	12 (3.5)	10 (3.5)	2 (4.0)	0.845
Previous PPI	34 (10.0)	28 (9.7)	6 (12.0)	0.610
Arterial hypertension	309 (90.9)	261 (90.0)	48 (96.0)	0.173
PHT	219 (64.4)	194 (66.9)	25 (50.0)	0.021 *
Diabetes mellitus	87 (25.6)	72 (21.2)	15 (30.0)	0.099
PAD	81 (23.8)	52 (17.9)	29 (58.0)	<0.001 *
CVD	63 (18.5)	54 (18.6)	9 (18.0)	0.917
Porcelain aorta	37 (10.9)	24 (12.0)	12 (24.0)	0.001 *
Hostile aorta	64 (18.8)	56 (19.3)	8 (16.0)	0.580
Previous RRT	1 (0.3)	1 (0.3)	0 (0.0)	0.678
CKD	131 (38.5)	116 (40.0)	15 (30.0)	0.180
COPD	80 (23.5)	67 (23.1)	13 (26.0)	0.656
Frailty	72 (21.2)	59 (20.3)	13 (26.0)	0.366
**Functional Data**				
STS score, %	2.7 ± 0.8	2.7 ± 0.8	2.5 ± 0.9	0.230
HAS-BLED score	2.9 ± 0.9	2.9 ± 0.9	2.8 ± 1.0	0.554
LVEF, %	55.1 ± 11.5	55.4 ± 11.5	52.7 ± 11.7	0.373
AVA, cm^2	0.8 ± 0.2	0.8 ± 0.2	0.8 ± 0.2	0.318
dPmax, mmHg	62.0 ± 23.3	61.5 ± 23.2	65.7 ± 23.9	0.306
dPmean, mmHg	39.0 ± 15.9	38.4 ± 15.6	43.2 ± 17.7	0.087

* $p < 0.05$; values are means ± SD, medians ± interquartile range, or n (%). AVA, aortic valve area; BMI, body mass index; CABG, coronary artery bypass graft; CAD, coronary artery disease; COPD, chronic obstructive pulmonary disease; CVD, cerebrovascular disease; dPmean/max, mean/max. transvalvular gradient; LVEF, Left ventricular ejection fraction; PCI, percutaneous coronary

intervention; PHT, pulmonary hypertension; PAD, peripheral artery disease; PPI, permanent pacemaker implantation; RRT, renal replacement therapy.
